# Acknowledging the Peer Reviewers of *Journal of Medical Radiation Sciences*, October 2024–September 2025

**DOI:** 10.1002/jmrs.70041

**Published:** 2025-11-13

The editorial review board, the Australian Society of Medical Imaging and Radiation Therapy (ASMIRT) and the New Zealand Society of Medical Imaging and Radiation Therapy (NZSMIRT) are grateful for the time and expertise of the following reviewers. Your contribution has been instrumental in producing a quality journal. We sincerely appreciate your dedication.

## Outstanding Reviewers (Contributed Five Reviews or More)

James Stanley

Amy Brown

Lynne Hazell

Gerhardus Koch

Sibusiso Mdletshe

Tracey Pieterse

Therese Gunn

Scott Jones

Angelina Piccolo

## The Following Reviewers Contributed Four Reviews

Karen Dobeli

Andrew Firman

Martin Ian Kamanda

Thandokuhle Khoza

Heather Lawrence

Kristie Matthews

Riaan van de Venter

Adrienne Young

## The Following Reviewers Contributed Three Reviews

Kamarul Abdullah

Luke Barclay

Vicki Braithwaite

Edel Doyle

Victoria Earl

Hesta Friedrich‐Nel

Georgia Halkett

James Hayes

Abel Karera

Tracy Kirkbride

Paul Lockwood

Clare McLaren

Renee Mineo

Alan Mui

Laura Murphy

Martin Necas

Bismark Ofori‐Manteaw

Warren Reed

Chi Yuan Wang

## The Following Reviewers Contributed Two Reviews

Mohamed Abuzaid

Kylie Auld

Nicole Robyn Badriparsad

Rachael Beldham‐Collins

Kylie Bradford

Bena Brown

Cameron Brown

Rachel Burton

Bernadette Byrne

Ernest Ekpo

James Elliott

Andrew England

Hafsa Essop

Mel Evans

Johnathan Hewis

Vanessa Hierl

Nurul Ismail

Yobelli Jimenez

Mary Job

Maeve Kearney

Toni Kelly

Andrew Kilgour

Ilona Lavender

Jens Loberg

Kevin London

Christina Malamateniou

Ellie Miller

Fairuz Mohd Nasir

Stuart More

Huong Nguyen

Julie Nightingale

Sagda Osman

Tracy Parker

Natalie Pollard

Claire Poole

Clare Singh

Adam Steward

John Thompson

Belinda Van der Merve

Bronwin Van Wyk

Francis Zarb

Nicole Zientara

## The Following Reviewers Contributed One Review

Ahmed Jibril Abdi

Dania Abu Awwad

Laura Adamson

Joanne Adlam

Theophilus Akudjedu

Christine Albantow

Azlinawati Ali

Katya Amadita

Nigel Anderson

Alyssa Asaro

Sally Ayesa

Marilyn Baird

Dmitry Beyder

N Borecky

Heidi Bowmast

Pippa Bresser

Alison Brown

Gemma Busuttil

Maddison Carroll

Michaela Cellina

Jacky Chen

Emma Cooper

Rob Davidson

Mikaela Dell'Oro

Naina Dhana

Dalia Dinham

Andrea Doubleday

Allison Dry

Kylie Dundas

Kirsten Elleray

Doaa Elwadia

Brendan Erskine

Elizabeth Forde

Clayton Frater

Bruce Goodwin

Kylie Grimberg

Emmanuel Gyan

Peter Hanna

Catriona Hargrave

Peter Hogg

Luisa E. Jacomina

Rodrigo Jaimovich

Rebecca Jude

John Kenny

Scott King

Drew Latty

Graeme Lazarus

Kim Lewis

Kelly Lloyd

Thulani Mabhengu

Tintswalo Mahlaola

Chandra Makanjee

Candice Mbaita

Clare McKenzie

James McNeil

Stefan Meng

Martin Mitchell

Mardhiyati Mohd Yunus

Lucinda Morris

Kathleen Naidoo

Afrooz Najafzadeh Abriz

Sharon Oultram

Vanessa Panettieri

Eric Pei Ping Pang

Shelley Park

Kunthi Pathmaraj

Malene Pedersen

Melanie Penfold

Melissa Rosemary Pillay

Jo‐Anne Pinson

Teresa Poon

Tania Poroa

Jonathan Loui Portelli

Selin Prasad

Robba Rai

Emma Rawlings

Tristan Reddan

John Robinson

Pamela Rowntree

Natalie Rozanec

Daniel Serra

Meegan Shepherd

Sumi Shrestha‐Taylor

Sierra Silverwood

Bev Snaith

Soundappan S. V. Soundappan

Aladdin Speelman

Tom Steffens

Kate Stewart

Zhonghua Sun

Kristie Sweeney

Rhonda‐Joy Sweeney

Linda Thebridge

Yu‐Feng Wang

Imelda Williams

Lee Wilton

Katrina Woodford

Nick Woznitza

## Peer Reviewer Recognition

JMRS reviewers can opt in to have their peer review contribution automatically added on Web of Science Reviewer Recognition Services (formerly Publons).

For more information please go to, https://authorservices.wiley.com/Reviewers/journal‐reviewers/recognition‐for‐reviewers/publons.html

## Peer Reviewer Resources

Are you new to conducting and writing peer reviews? Check out these free peer reviewer guidelines and free self‐directed learning modules.

Wiley Journal Reviewers

https://authorservices.wiley.com/Reviewers/journal‐reviewers/index.html

Wiley Researcher Academy: All you need to know to become an effective peer reviewer

https://www.wileyresearcheracademy.com/p/all‐you‐need‐to‐know‐to‐become‐an‐effective‐peer‐reviewer

Web of Science Academy

https://clarivate.com/webofsciencegroup/solutions/web‐of‐science‐academy/

